# Medical Opinion and Movement

**Published:** 1906-07-28

**Authors:** 


### Medical Opinion and Movement.

Mr. Alfred Beit was probably the ablest, and certainly one of the most lovable, of the great financiers of the century. He was always so clear-headed and intelligent, so kind-hearted and so just, that it is recorded that when his death was announced, there was not a single man connected with his enterprises who did not experience a sense of personal loss. Mr. Beit was not understood by the English people during the more active portion of his career, but his will, like that of the late Cecil Rhodes, has an educational value which must make its influence felt for good as an example to wealthy men and women throughout the world.

We have dwelt upon the personal attributes of the late Mr. Alfred Beit because they must have an interest for the medical profession, from the circumstance that his will has recognised the supreme value of science in medicine, and must therefore exercise a permanent influence upon the cure of disease. He has supplemented a former munificent gift to the Institute of Medical Sciences Fund at the University of London by a further £25,000. It would be of immense value to the race if wealthy men could be brought to understand that, providing money in sufficient quantities for scientific research were forthcoming, men and women might speedily be delivered from the ravages of tuberculosis, cancer, and other diseases by discoveries which would eradicate these diseases for all time. Discovery is largely a question of money, and abundance of money for research should be speedily found. Mr. Alfred Beit's name should be held in grateful memory by all who know most about hospitals, from the wisdom he displayed in giving £20,000 to King Edward's Hospital Fund and £20,000 to Guy's Hospital, which latter, when everything is taken into consideration, may probably be regarded as the greatest of English medical schools.

We are glad to record an excellent piece of philanthropic work for children just completed by the Vienna Society for the erection of seaside hospitals for children. The object of the Society was to provide for thousands of tuberculous children by supplying bathing accommodation for them on the Adriatic, where the water is heavily charged with iodine. The Vienna Society has founded, erected, and opened three hospitals, with which convalescent sanatoria are connected, and it has placed them under the management of a council, of which Professor Monti, the chief of the Vienna Children's Hospital system, is the Chairman, which consists of representatives of the Vienna municipality, of the Society, and of the medical officers of the hospitals of Vienna. It is in contemplation to provide 600 beds, and the movement is memorable because it is practically the first time that money has been supplied in Austria in large quantities from voluntary contributions. It is a notable fact, and reflects great honour on the men who are responsible for the movement, that these hospitals and sanatoria have been erected at a cost of about £150 per bed.

In England seaside provision for tuberculous children is mainly dependent upon voluntary effort. Some of the more intelligent boards of guardians have made provision for the reception of these cases in buildings at the seaside, but this movement has not been developed through the Poor-law system as it might and ought to be. Voluntary effort has provided convalescent homes for children at Brighton, Eastbourne, New Brighton, Rhyl, Thanet, Southsea, Torquay, and Weymouth. The most recent movement of this kind is connected with the Children's Sanatorium at Holt, on the east coast of Norfolk, where a temporary home has been opened for children in the early stages of tuberculosis. The terms of admission are rather high (25s. a week inclusive), but it is intended to give assistance, as far as funds permit, in cases recommended by the Invalid Children's Aid Association. We welcome the Holt Sanatorium because it is calculated to do a useful work and to be well administered under the auspices of the Invalid Children's Aid Association, one of the best and most useful organisations for children in the country.

Reverting to the present position of the British Medical Association, it is interesting to note, as Dr. Mackenzie Johnston points out, that the income of the Association, which was £25,385 in 1886, had increased in 1905 to £50,107—that is to say, the income has practically doubled in twenty years. Unfortunately the expenditure has not only doubled, but has, in fact, increased by 133 per cent., so that, unless some change of policy is speedily adopted, the consequences may be disastrous. The larger increase in expediture has arisen since the new constitution was adopted, and it is properly contended that the present pace must be regulated and the expenditure reduced. The Association owes grateful thanks to Dr. T. Arthur Helme, of Manchester, for circulating his memorandum, the point of which is thus tersely stated: "Were it not for the prudence of our predecessors we should this year have exceeded our income by £2,708." What the Association needs is a speed limit, and it must be satisfactory to the members to note that the Council seems fully alive to the urgent necessity for the application of such a break, for the recommendation as to the charter was adopted by a large majority at the last meeting. Members will do well to remember, with Dr. Johnston, that "the healthiest and most lasting of reforms comes slowly and gradually after mature consideration."

